# In Reply to "The Relationship Between Erythropoietin Resistance and Antibody Response to Hepatitis B Vaccine in Hemodialysis Patients"

**DOI:** 10.5812/numonthly.14330

**Published:** 2013-11-13

**Authors:** Seyfollah Beladimusavi, Fatemeh Hayati

**Affiliations:** 1 Chronic Renal Failure Research Center, Department of Internal Medicine, Faculty of Medicine, Ahvaz Jundishapur University of Medical Sciences, Ahvaz, IR Iran; 2 Department of Internal Medicine, Faculty of Medicine, Jundishapur University of Medical Sciences, Ahvaz, IR Iran

**Keywords:** Erythropoietin, Hepatitis B, Renal Dialysis, Vaccines

***Dear Editor,***

We read with great interest the article by Baris Afsar entitled "The relationship between erythropoietin resistance and antibody response to hepatitis B vaccine in hemodialysis patients" published in your valuable journal. He analyzed the relationship between anti-HBs response and erythropoietin (EPO) resistance among patients undergoing maintenance hemodialysis and demonstrated that EPO resistance is negatively associated with both anti-HBs levels and seroconversion status among these patients (1).

Hyporesponsive to EPO therapy or EPO resistance and effectiveness of hepatitis B virus (HBV) vaccination in patients with end-stage renal disease (ESRD) are important issues among these patients. Unfortunately, EPO resistance is relatively common and it may be an important clinical observation since a hyporesponsive to EPO therapy and need to higher doses of EPO may be associated with increased mortality, an effect that persists after adjustment for the usually lower hematocrit in such patients (2-4).

A variety of factors may cause patients to be relatively resistance to EPO including absolute iron deficiency which may be due to external blood losses, bone disease due to secondary hyperparathyroidism or the accumulation of aluminum in bone, chronic inflammation, the administration of angiotensin converting enzyme inhibitors and/or angiotensin II receptor antagonists, unsuspected hematologic disorders, development of pure red cell aplasia and etc. (5-9).

Compared to the overall effectiveness of HBV vaccination in patients without renal failure (a response rate of over 90 percent), the effect of this vaccination is reduced among patients with ESRD because of the general suppression of the immune system associated with uremia. It appears that only 50 to 60 percent of these patients develop antibodies following HBV vaccination. The rate of response is higher among patients with chronic kidney disease not requiring dialysis, suggesting that the immune response is positively associated with the degree of renal function (10-12).

Although it could be possible and the results of Afsar study suggested that EPO resistance was negatively associated with anti-HBs levels, however, the study has limitations including retrospective design, small number of patients, not available reliable information about the brand of vaccines and the results of the study cannot prove a cause and effect relationship between EPO resistance and anti-HBs levels. Therefore, further prospective researches with a larger number of patients are needed to highlight the underlying mechanisms of EPO resistance and relationship between it and anti-HBs levels and seroconversion status among HD patients.
